# Propofol Causes Vasodilation *In Vivo* via TRPA1 Ion Channels: Role of Nitric Oxide and BK_Ca_ Channels

**DOI:** 10.1371/journal.pone.0122189

**Published:** 2015-04-01

**Authors:** Sayantani Sinha, Pritam Sinharoy, Ian N. Bratz, Derek S. Damron

**Affiliations:** 1 Department of Biological Sciences, Kent State University, Kent, Ohio, United States of America; 2 Department of Integrative Medical Sciences, Northeast Ohio Medical College, Rootstown, Ohio, United States of America; St. Joseph's Hospital and Medical Center, UNITED STATES

## Abstract

**Background:**

Transient receptor potential (TRP) ion channels of the A1 (TRPA1) and V1 (TRPV1) subtypes are key regulators of vasomotor tone. Propofol is an intravenous anesthetic known to cause vasorelaxation. Our objectives were to examine the extent to which TRPA1 and/or TRPV1 ion channels mediate propofol-induced depressor responses *in vivo* and to delineate the signaling pathway(s) involved.

**Methods:**

Mice were subjected to surgery under 1.5–2.5% sevoflurane gas with supplemental oxygen. After a stable baseline in mean arterial pressure (MAP) was achieved propofol (2.5, 5.0, 10.0 mg/kg/min) was administered to assess the hemodynamic actions of the intravenous anesthetic. The effect of nitric oxide synthase (NOS) inhibition with L-NAME and/or calcium-gated K^+^ channel (BK_Ca_) inhibition with Penetrim A (Pen A), alone and in combination, on propofol-induced decreases in mean arterial pressure were assessed in control C57Bl/6J, TRPA1-/-, TRPV1^-/-^ and double-knockout mice (TRPAV^-/-^).

**Results:**

Propofol decreased MAP in control mice and this effect was markedly attenuated in TRPA1^-/-^ and TRPAV^-/-^ mice but unaffected in TRPV1^-/-^mice. Moreover, pretreatment with L-NAME or Pen A attenuated the decrease in MAP in control and TRPV1^-/-^ mice, and combined inhibition abolished the depressor response. In contrast, the markedly attenuated propofol-induced depressor response observed in TRPA1^-/-^ and TRPAV^-/-^ mice was unaffected by pre-treatment with Pen A or L-NAME when used either alone or in combination.

**Conclusion:**

These data demonstrate for the first time that propofol-induced depressor responses *in vivo* are predominantly mediated by TRPA1 ion channels with no involvement of TRPV1 ion channels and includes activation of both NOS and BK_Ca_ channels.

## Introduction

Transient receptor potential ankyrin subtype 1 (TRPA1) and transient receptor potential vanilloid 1 (TRPV1) are ligand-gated cation channels that play pivotal roles in regulating pain and inflammatory pathways [1–4]. Recent evidence has emerged demonstrating a prominent role of TRPV1 in the regulation of vascular function and while studies investigating the role of TRPA1 in the vasculature are controversial, some recent reports have confirmed that the TRPA1 channel is involved in regulating vasomotor tone [5–8]. Moreover, upregulation of TRPV1 expression has been implicated in mediating pathophysiological conditions related to hypertension and diabetes [9–13] while studies investigating the role of TRPA1 in the pathophysiological setting are lacking.

Studies from our laboratory and others have shown that these channels communicate with each other [14–17]. Moreover, it has been demonstrated that the functionality of one channel can be dependent on the presence of the other [18–19] and evidence exists confirming that these channels are capable of assembling into homo- or heterotetrameric complexes [19–21]. However, most of these studies have been performed in neurons and expression systems and no studies have examined and identified crosstalk between these channels in the regulation of vascular tone.

Propofol is an intravenous anesthetic known to cause vascular relaxation [22–25]. Recent evidence has demonstrated that propofol activates TRPA1 and to some extent TRPV1 in sensory neurons and heterologous expression systems [26–28]. In fact, a variety of anesthetic agents have been shown to interact with TRP channels and either activate or sensitize the channel [29–31]. Moreover, our laboratory has demonstrated that propofol restores TRPV1 sensitivity to agonist stimulation via a TRPA1-dependent pathway involving activation of protein kinase C epsilon demonstrating cross-talk between the channels in sensory neurons [14,15]. However, the extent to which either of these channels involved in mediating propofol-induced vasodilation has not been examined.

In the current *in vivo* study, we tested the hypothesis that propofol causes vasodilation, via a TRPA1-dependent pathway. Moreover, we also tested the hypothesis that the propofol-induced depressor response is mediated by an endothelial nitric oxide synthase (eNOS) pathway involving activation of large conductance, Ca^2+^ activated K^+^ channels (BK_Ca_) channels. The major finding is that propofol induces depressor responses *in vivo* that are primarily mediated by TRPA1 with little if any TRPV1 involvement. In addition, inhibition of eNOS with L-Nitro Arginine Methyl Ester (L-NAME) or BK_Ca_ channels with Penitrem A (Pen A) attenuated the propofol-induced depressor response in control mice, and combination of both inhibitors virtually abolished the response. Similar effects were observed in TRPV1^-/-^ mice. In contrast, inhibition of eNOS and/or BK_Ca_ channels had no additional effect on the already markedly attenuated propofol-induced depressor response in TRPA1^-/-^ or TRPAV^-/-^ double knockout mice unlike that observed in control or TRPV1^-/-^ mice. Our current findings indicate that TRPA1 is the predominant player in the propofol-induced depressor response observed *in vivo* and further indicate that an eNOS-dependent and BK_Ca_-dependent pathway are involved in mediating the response.

## Materials and Methods

All experiments were conducted with the approval of Institutional Animal Care and Use Committee at Kent State University and NEOMED in accordance with The National Institutes of Health Guidelines for the Care and Use of Laboratory Animals. Mice breeding pairs were purchased from Jackson Labs (Bar Harbor, ME) and were bred in the animal facility of NEOMED. Mice were housed in a room with a 12:12-h light-dark cycle and maintained with constant temperature and continuous access to food and water. Experiments were performed in 8–12 week old males of C57Bl6, TRPA1^-/-^, TRPV1^-/-^ mice and in the double knockout TRPAV^-/-^ mice.

### Generation of TRPAV^-/-^ mice

TRPA1^-/-^ and TRPV1^-/-^ mice were purchased from Jackson labs. TRPA1^-/-^ females were bred with TRPV1^-/-^ mice to obtain TRPAV heterozygotes (TRPA1^+/-^, TRPV1^+/-^). These heterozygotes were then bred to obtain the double knockouts (TRPAV^-/-^).

### Genotyping

Genotyping was performed by PCR using genomic DNA. Genomic DNA was obtained by alkaline lysis of tailpieces. The TRPA1 transgene was detected using the primers 5’-TCCTGCAAGGGTGATTGCGTTGTCTA-3’ (WT forward) and 5’-TCATCT GGGCAACAATGTCACCTGCT-3’ (WT reverse) and 5’-CCTCGAATCGTGGATCCACTAGTTCTAGAT-3’ (mutant forward) and 5’-GAGCATTACTTACTAGCATCCTGCCGTGCC-3’ (mutant reverse) primers. Similarly the TRPV1 transgene was detected using the primers 5’-CCTGCTCAACATGCTCATTG-3’ (WT forward), 5’-TGGATGTGGAATGTGTGCGAG-3’ (mutant forward) and 5’-TCCTCATGCACTTCAGGA AA-3’(common reverse) primers.

### Measurement of Mean Arterial Pressure

Mice were subjected to surgery under 1.5–2.5% sevoflurane gas with supplemental oxygen using a Veterinary Anesthesia and Monitoring Device. Mice were placed on a temperature-controlled table where the core temperature was maintained via a rectal probe at 37⁰C. The right jugular vein and femoral artery were cannulated as previously described [10] with a high-fidelity microtip transducer catheter connected to a data acquisition system (PowerLab ML820, ADInstrument, Colorado Springs) through a pressure interface unit (Millar Instrument, Transducer Balance, TCB 600) to measure systolic, diastolic, pulse pressure, MAP and heart rate (HR). MAP data were analyzed using AD-Instrument Chart v5.1.2 software. Following surgery, mice were given a bolus injection of the ganglionic blocker hexamethonium (5 mg/kg; Sigma, St. Louis, MO) to eliminate reflex adjustments and to focus on the primary vascular actions of propofol. Initial studies were performed to determine the effects of continuous infusion of propofol (2.5–10 mg/kg/min) administered at the rate of 20 μl/min for 4 min after a stable base-line of mean arterial pressure was achieved (approx. one hour after sevoflurane anesthesia) in the presence and absence of the various interventions (10 min pre-treatment) in order to assess the hemodynamic action of the anesthetic. The concentrations of propofol used in the current study were similar to those used by others and are well within the clinically relevant range [32]. The Intralipid vehicle had no effect on MAP. Hemodynamic response curves were performed in all groups to propofol in the presence and absence of the antagonists L-NAME (100 mg/kg/min) and/or Pen A (50μg/kg/min). Ten minutes elapsed following each inhibitor before propofol infusion began to allow for MAP to stabilize. Pressures and/or HR were continuously recorded throughout the experiment.

### Drugs and Chemicals

All drugs were purchased from Sigma Chemicals (St. Louis, MO, USA) and dissolved in distilled water as concentrated stock solutions unless otherwise stated. Propofol stock solution (10 mg/ml, injectable emulsion) was purchased from Cleveland Clinic Foundation.

### Data Analysis and Statistics

The mean base-line values of HR and MAP were taken at 30 seconds before the infusion of drugs. The largest value after infusion of drug was taken as the maximum MAP. The percent change in MAP or relaxation was calculated using the formula: % change = (Maximum-Base-line)/ Baseline times 100. Data are expressed as means ± SEM. Statistical comparisons were performed using one-way analysis of variance followed by pairwise multiple comparison procedures such as Tukey or Dunn test as appropriate. For statistical analyses, Sigma Plot 11.0 software (Systat Software, San Jose, CA) was utilized. A value of p < 0.05 was considered statistically significant.

## Results

### Baseline hemodynamics of control, TRPV1^-/-^, TRPA1^-/-^ and TRPAV^-/-^ mice

Table 1 depicts the baseline hemodynamic data obtained from all mice groups after addition of HEX but prior to any other intervention. Body weight is represented as follows (in g): TRPA1^-/-^ (29.0 ± 1.32), TRPAV^-/-^ (28 ± 2); TRPV1^-/-^ (27 ± 0.7) and control (25 ± 0.6). Base-line heart rate (HR) was significantly higher in TRPA1^-/-^ (580 ± 39 beats/min) and TRPAV^-/-^ (543 ± 16 beats/min) compared with controls (400 ± 14.5 beats/min). Baseline MAP was recorded before any perturbation. TRPAV^-/-^ mice (94 ± 3.13 mm Hg) were significantly hypertensive compared with TRPV1^-/-^ (82 ± 1.05 mm Hg) and control mice (81.8 ± 2.8 mm Hg). There was no significant change in HR following administration of propofol in control mice (410 ± 15 beats/min) compared to basal HR. Similarly, there was no effect of propofol on HR in TRPA1^-/-^, TRPV1^-/-^ and TRPAV^-/-^ compared to basal HR for each group of mice. Inhibition of TRPA1 channels with HC-0300301 caused a slight increase in mean arterial pressure (2.6 ± 2.8 mm Hg) with no significant change in heart rate (data not shown).

**Table 1:** Baseline Hemodynamics.

** **	**Control**	**TRPA1** ^-/-^	**TRPV1** ^-/-^	**TRPAV** ^-/-^
**Body Weight (g)**	**25 ± 0.6**	**29 ± 1.32**	**27 ± 0.7**	**28 ± 2**
**Basal heart rate, beats/min**	**400 ± 14.5**	**580 ± 39** *	**471 ± 27**	**543 ± 16** *
**Systolic blood pressure, mmHg**	**96.8 ± 2.5**	**109 ± 1.7** *	**99 ± 1.08**	**112 ± 3.02** *
**Diastolic blood pressure, mmHg**	**69 ± 3.2**	**76 ± 1.2**	**66 ± 2.5**	**78 ± 2.7** *
**MAP, mmHg**	**81.8 ± 2.8**	**90 ± 1.0**	**82 ± 1.05**	**94 ± 3.13** *

Values are represented as Mean ± SE and were obtained after addition of hexamethonium. Body weights were recorded before anesthesia [Control: n = 26; TRPA1 ^-/-^: n = 12; TRPV1^-/-^: n = 12 and TRPAV^-/-^: n = 12]. MAP = Mean Arterial Pressure.

(*) denotes significance from control p<0.05.

### Effect of Propofol on MAP: Role of TRPA1 and TRPV1

**Fig 1:**
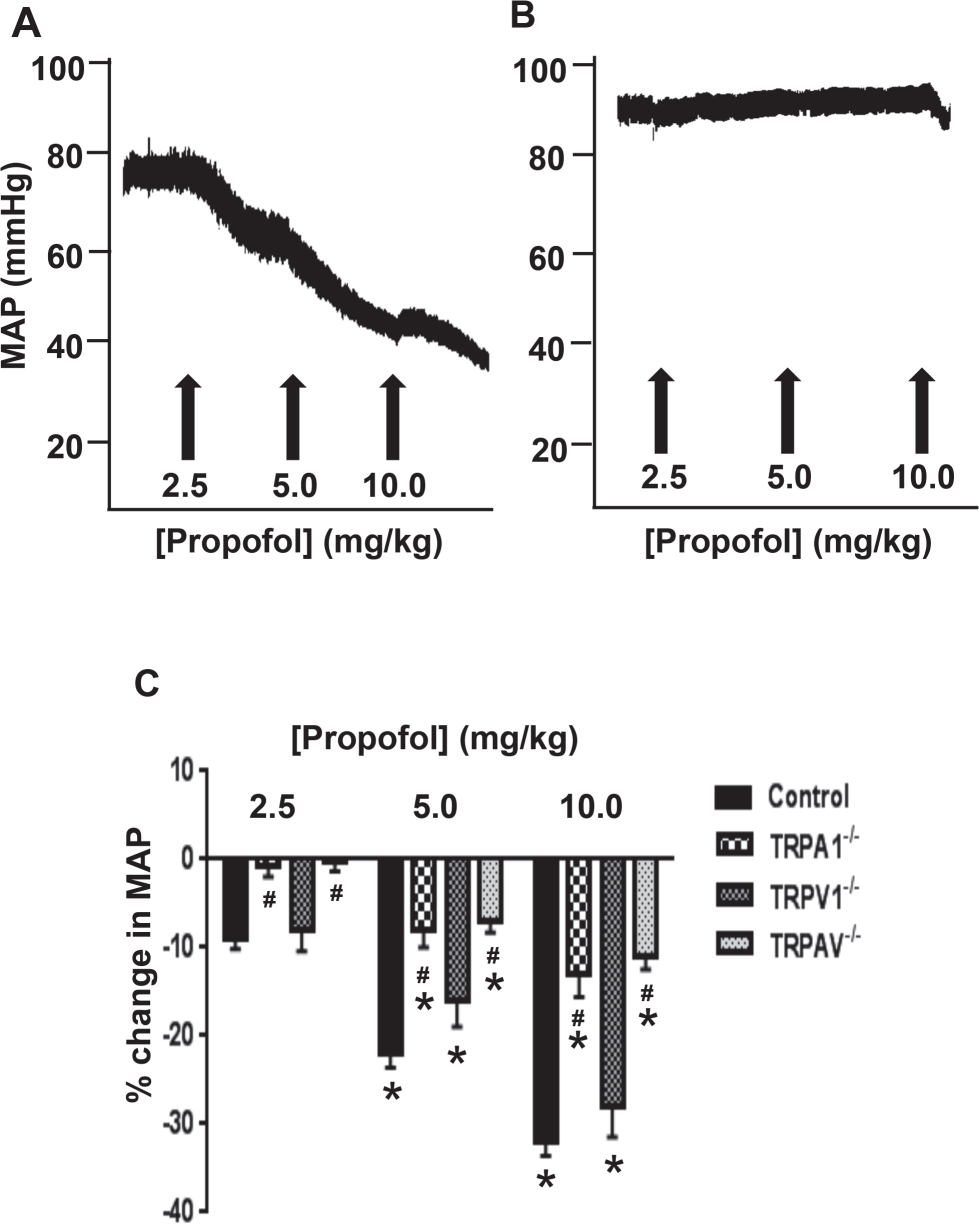
Effect of Propofol on MAP. Panel **A** and **B** shows representative traces depicting the effect of propofol on MAP in control and TRPA1^-/-^ mice. Panel **C** depicts summarized data depicting the effect of propofol on MAP in control (n = 26), TRPA1^-/-^ (n = 12), TRPV1^-/-^ (n = 12) and TRPAV^-/-^ mice (n = 12). Data are means ± SEM * = *P*<0.05 vs. baseline. # *P*<0.05 vs. control.

Propofol decreased MAP in a dose-dependent manner (Fig 1A and 1C) in control mice that was markedly attenuated in TRPA1^-/-^ (Fig 1B) and TRPAV^-/-^ mice (Fig 1C). However, the propofol-induced decrease in MAP in TRPV1^-/-^ mice was similar to that observed in control mice (Fig 1C).

### Effect of NOS and/or BK_Ca_ Channel Inhibition on Propofol-Induced Depressor Responses

**Fig 2:**
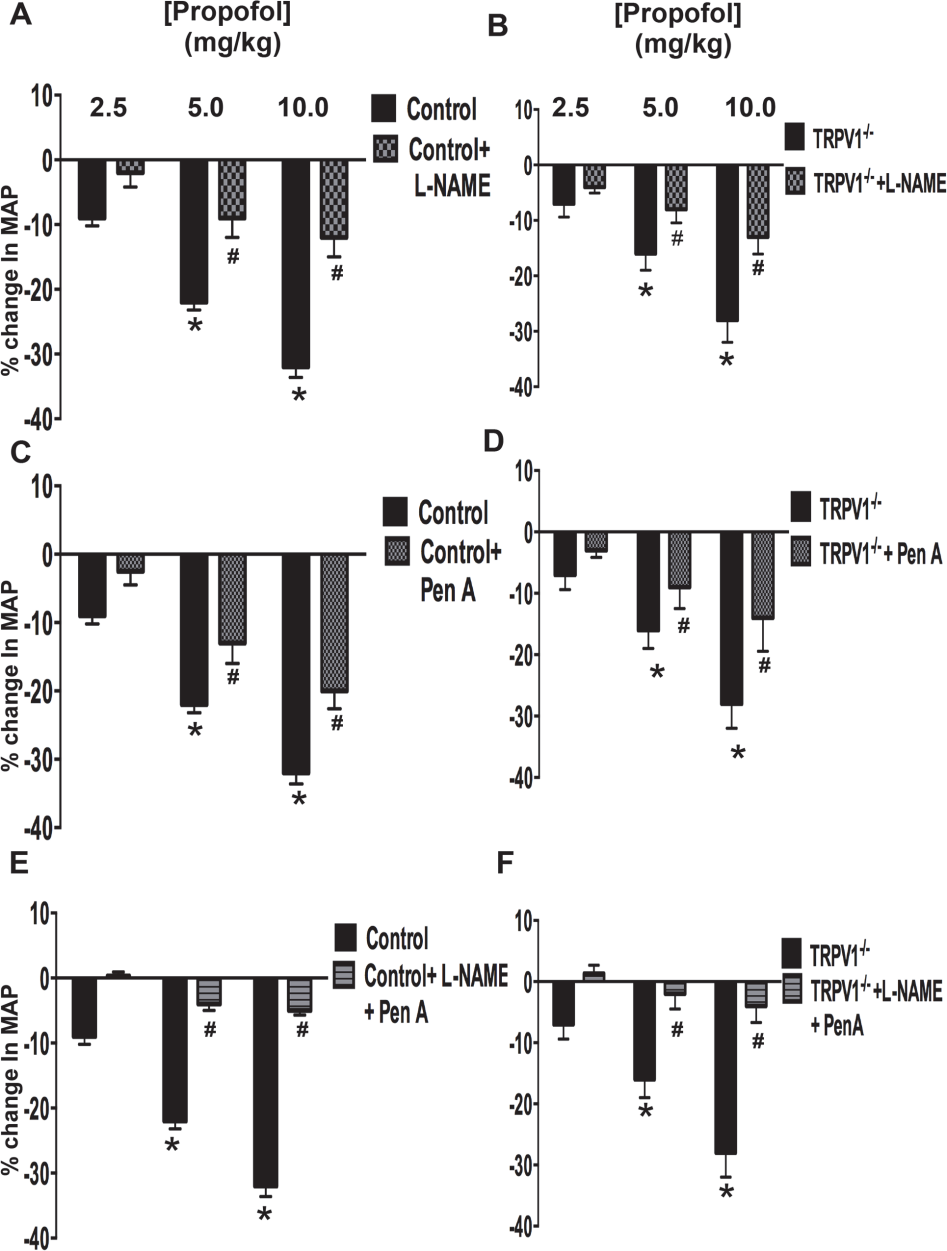
Propofol induced changes in MAP in Control and TRPV1^-/-^ mice: Panels A and B: Summarized data depicting the effect of L-NAME (100 mg/kg/min) on propofol-induced changes in MAP in control and TRPV1^-/-^ mice. Panels **C** and **D:** Summarized data depicting the effect of Pen A (50 ug/kg/min) on propofol-induced changes in MAP in control and TRPV1^-/-^ mice. Panels **E** and **F:** Summarized data depicting the effect of L-NAME and Pen A in combination on propofol-induced changes in MAP in control and TRPV1^-/-^ mice. Data are means ± SEM. *P<0.05 compared to baseline. #*P*<0.05 compared to control. *n* = 6 mice in each group.

**Fig 3:**
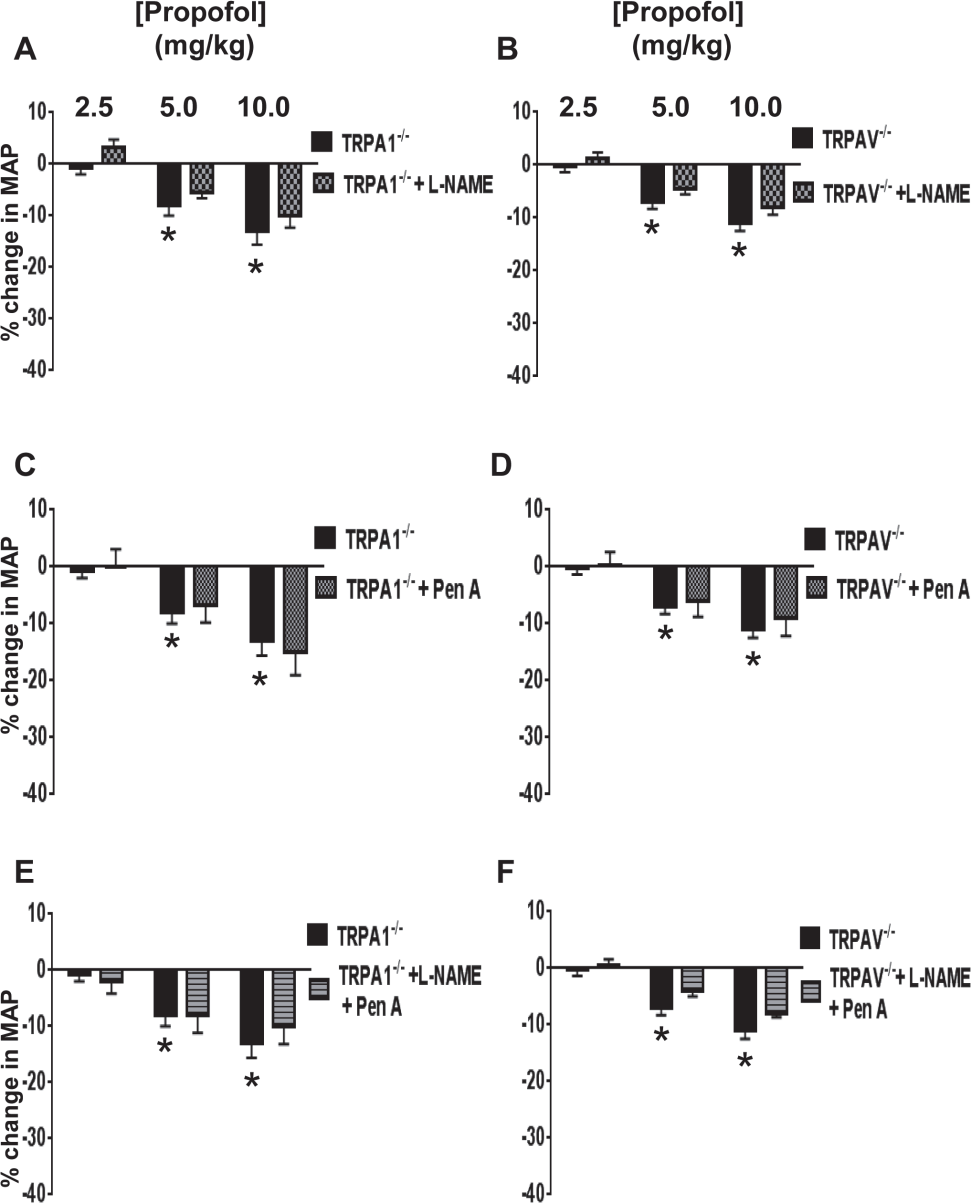
Propofol induced changes in MAP in TRPA1^-/-^ and TRPAV^-/-^ mice: Panels A and B: Summarized data depicting the effect of L-NAME (100 mg/kg/min) on propofol-induced changes in MAP in TRPA1^-/-^ and TRPAV^-/-^ mice. Panels **C** and **D:** Summarized data depicting the effect of Pen A (50ug/kg/min) on propofol-induced changes in MAP in TRPA1^-/-^ and TRPAV^-/-^ mice. Panels **E** and **F:** Summarized data depicting the effect of L-NAME and Pen A in combination on propofol-induced changes in MAP in control and TRPA1^-/-^ and TRPAV^-/-^ mice. Data are means ± SEM. *P<0.05 compared to baseline. *n* = 6 mice in each group.

To determine the extent to which NO and BK_Ca_ channels are involved in mediating the propofol-induced depressor response *in-vivo*, (L-NAME; 100mg/kg) and/or Pen A (50μg/kg) were used alone and in combination to inhibit eNOS and BK_Ca_ channels, respectively. Administration of L-NAME significantly increased MAP (13 ± 3.1* mmHg) yet markedly attenuated the propofol-induced depressor response at all concentrations of propofol in control mice (Fig 2A). Because there is an increase in MAP after L-NAME administration in all groups, the % change in MAP following the various interventions was calculated from the newly established baseline value following L-NAME administration in all groups. In addition, administration of Pen A had no significant effect on baseline MAP (-1 ± 3.5 mmHg) yet blunted the propofol-induced depressor response in control mice (Fig 2C). Moreover, the combination of L-NAME and Pen A significantly increased baseline MAP (difference from base-line: 14.4 ± 3.2 mmHg) and resulted in an additive effect on attenuation of the propofol-induced depressor response when compared to L-NAME or Pen A alone (Fig 2E). Similar results were observed in TRPV1^-/-^ mice (Fig 2B, 2D and 2F). To further delineate a connection between TRPA1, NO and BK_Ca_ channels in mediating the propofol-induced depressor response *in vivo*, L-NAME and Pen A were utilized alone and in combination in TRPA1^-/-^ and TRPAV^-/-^ mice. As noted in Fig 1C, the propofol-induced depressor response was markedly reduced in TRPA1^-/-^ and TRPAV^-/-^ mice. The presence of L-NAME or Pen A, either alone or in combination, had no significant effect on the propofol-induced depressor response in TRPA1^-/-^ (Fig 3A, 3C and 3E) or TRPAV^-/-^ mice (Fig 3B, 3D and 3F).

## Discussion

This is the first study to investigate the role of TRP ion channels in mediating propofol-induced depressor responses *in vivo*. Numerous studies have identified that propofol has profound vasodilatory properties both *in vivo* [22,33] and *in vitro* [25,34–37]. Moreover, anesthetics have been shown to activate as well as modulate TRPA1 and/or TRPV1 ion channel sensitivity to agonist activation in sensory neurons and heterologous expression systems [14,15,26,27,29,30]. However, a link between anesthetics and TRPA1 or TRPV1 activation in the modulation of vasomotor tone *in vivo* has yet to be established. The novel findings of the present study are that propofol-induced depressor responses *in vivo* are mediated, at least in part, by TRPA1 but not TRPV1 channels. Moreover, inhibition of eNOS or BK_Ca_ channels individually both markedly attenuate the propofol-induced depressor response whereas combined inhibition virtually abolishes the vasodilator effect. In addition, the effect of eNOS and BK_Ca_ inhibition is lost in TRPA1^-/-^ and TRPAV^-/-^ mice.

### Effects of Propofol on TRPA1 and TRPV1 Activation

The effects of propofol on TRPA1 and/or TRPV1 are controversial. Matta et al [26] demonstrated that clinical concentrations of propofol (and other general anesthetics) excite sensory neurons by selectively activating TRPA1 with no effect on TRPV1 (similar results obtained in TRPA1 or TRPV1-transfected HEK 293 cells). Further, propofol-induced pain-related responses in mice were abolished in TRPA1-null animals and unaffected in TRPV1 null animals [26]. Fischer et al [27] demonstrated a propofol-induced activation of TRPA1 and TRPV1 in transfected HEK 293 cells however in dorsal root ganglion neurons, propofol-induced activation correlated better with TRPA1 rather than TRPV1. Nishimoto et al [45] demonstrated a propofol-induced activation of human and mouse TRPA1 whereas they did not observe propofol-evoked TRPV1 activation and the ability to activate mouse TRPV1 was very small. The same group demonstrated that propofol still caused an increase in intracellular Ca^2+^ in neurons obtained from TRPA1/TRPV1 double knockout mice as a result of GABA receptor activation leading to activation of T- and L-type channels and Fischer et al [27] similarly also reported a propofol-induced GABA receptor activation as well, albeit to a much lesser extent, than that of TRPV1 or TRPA1.

### Effect of Propofol on MAP: Role of TRPA1 and TRPV1

The mechanism of action of propofol on the vasculature is controversial and may involve direct modulation of vascular tone in an endothelium-dependent or independent manner depending on the species (rat, porcine and human) and/or vascular bed (thoracic and coronary) from which the arterioles were obtained [24,25,38,39,40]. Specifically, endothelial denuding [37,39,40] and e-NOS inhibition [25] resulted in sustained dilation to propofol, whereas [24] demonstrate a role for BK channels in the response. Although TRPA1 channels have been identified as important modulators of vasomotor tone *in vivo* and *in vitro*, [5,7]^,^ no studies have attempted to link the vasodilatory properties of propofol to activation of TRP ion channels in the vasculature. Our baseline measurements indicate that the elevated MAP and heart rate in the TRPA1 and double knockout are likely due to the global knockout and not due to presence of sevoflurane since all animals were administered sevoflurane. Preliminary data indicate that inhibition of TRPA1 with HC-030031 in wild-type mice resulted in slight increase in MAP (<5mm Hg) suggesting a modest tonic basal activity of the channel. In the current study, we observed a dose dependent decrease in MAP following administration of clinically relevant concentrations of propofol [32,40] in control mice. *In vivo*, the propofol-induced decrease in MAP was unaltered in TRPV1^-/-^ mice, but markedly attenuated (>50%) in TRPA1^-/-^ and TRPAV^-/-^ mice. In fact, the depressor response to propofol was virtually abolished in the TRPA1^-/-^ and TRPAV^-/-^ mice at the lowest concentration of propofol (2.5 mg/kg) tested which represents a clinically relevant dose typically used for induction and maintenance of anesthesia (2–2.5 mg/kg). Therefore, our data indicate for the first time that TRPA1 channels play a predominant role in mediating the propofol-induced depressor response observed *in vivo*. Although TRPA1 channels do not mediate the entire depressor response observed with higher concentrations of propofol, our future studies are aimed at delineating additional mechanisms involved in mediating the depressor effect. We next sought to determine the downstream pathways involved in mediating the propofol-induced decrease in MAP observed *in vivo*.

### Effect of NOS and/or BK_Ca_ Channel Inhibition on Propofol-Induced Depressor Responses

In order to address the downstream signaling pathways, we first assessed the role of NO in mediating the propofol-induced depressor response in all four groups of mice. Our studies indicate that inhibition of eNOS with L-NAME prior to administration of propofol markedly attenuated the propofol-induced depressor response in control and TRPV1^-/-^ mice, an effect that was not observed in TRPA1^-/-^ or TRPAV^-/-^ mice. These data indicate that stimulation of TRPA1, but not TRPV1 channels results in activation of eNOS and production of NO that mediates approximately 50% of the propofol-induced depressor response *in vivo*. Recent findings of Wu et al which demonstrated TRPV1 and BK channels have the ability to form a signaling complex [41]. This thus could be a compensatory mechanism for the loss of TRPA1 and/or TRPV1. The loss of TRPA1 would potentially allow for the formation of more TRPV1-BK complexes and vice versa for the loss of TRPV1. Moreover loss of either TRPA1 or TRPV1 may lead to altered NO signaling. Finally, we previously demonstrated that TRPA1 is vital for re-sensitization of previously desensitized TRPV1 [15] thus loss of TRPA1 channels could result in prolonged desensitization of endothelial TRPV1 resulting in blunted endothelial-dependent TRPV1 signaling. There is a previous report that NO mediates propofol-induced peripheral vasodilation in chronically instrumented dogs, [23] as well as a report that propofol hyperpolarizes rat mesenteric vascular smooth muscle *in situ* via a NOS-dependent pathway [42]. Moreover, several *in vitro* studies have also implicated NO as the mediator of propofol-induced vasodilation in isolated mesenteric [34] and distal coronary arterial rings [25]. However, no study to our knowledge has demonstrated a role for TRPA1 in mediating propofol-induced, NOS-dependent depressor responses *in vivo* or *in vitro*.

We next assessed the role of BK_Ca_ channels in mediating the effects of propofol. Similar to our findings with L-NAME, inhibition of BK_Ca_ channels with Pen A prior to administration of propofol markedly attenuated the propofol-induced depressor response in control and TRPV1^-/-^ mice, an effect that was not observed in TRPA1^-/-^ or TRPAV^-/-^ mice. These data indicate that stimulation of TRPA1, but not TRPV1 channels results in activation of BK_Ca_ channels that mediates about 40–50% of the propofol-induced depressor response *in vivo*. Moreover, combination of L-NAME and Pen A pretreatment prior to administration of propofol resulted in an additive effect that virtually abolished the propofol-induced depressor response *in vivo* suggesting the two pathways may be acting in parallel downstream of TRPA1 activation. A previous study also implicated BK_Ca_ channels in mediating the relaxing effect of propofol on coronary arteries *in vitro* [24] and another study demonstrated that NO and BK_Ca_ channels were involved in mediating disruption of TRPV1-mediated coupling of coronary blood flow to cardiac metabolism in diabetic mice *in vivo* [10]. To our knowledge, this is the first study to link NO and BK_Ca_ channels to propofol-induced, TRPA1-mediated depressor responses *in vivo*.

### Summary and Conclusion

**Fig 4:**
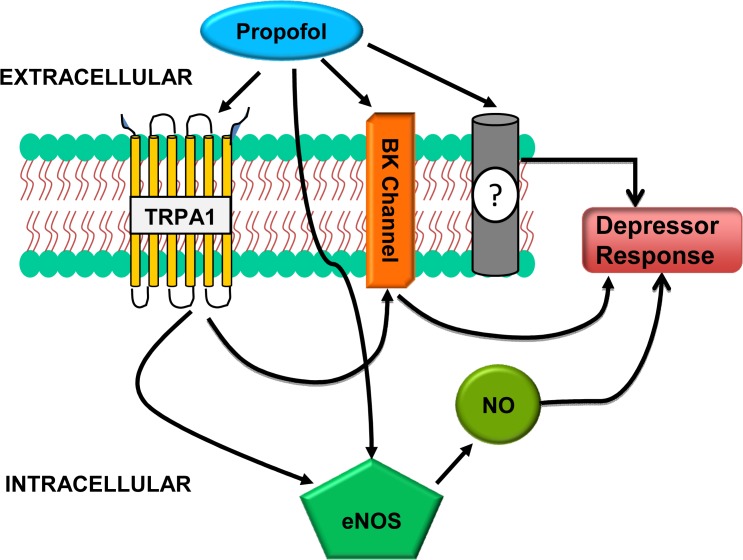
A schematic representation showing the proposed mechanisms by which propofol causes vasodepressor responses *in-vivo*.

Our data suggest that TRPA1, at least in part, but not TRPV1 is involved in mediating the propofol-induced depressor response and that NO production and BK_Ca_ activation are also involved downstream of TRPA1. In addition, NOS inhibition and BK_Ca_ inhibition had an additive effect in control and TRPV1^-/-^ mice but virtually no additional effect in TRPA1^-/-^ or TRPAV^-/-^ mice suggesting TRPA1 stimulation is coupled to both NOS activation and BK_Ca_ activation and the pathways appear to be operating in parallel (Fig 4). Moreover, because the depressor response induced by propofol is not completely abolished when TRPA1 and/or TRPV1 are deleted, yet combined inhibition of eNOS and BK_Ca_ channels abolishes the effect, suggests propofol may be directly interacting with eNOS and/or BK_Ca_ channels *in vivo* (Fig 4). Alternatively, other channels and/or mediators may also be involved. Some previous reports have demonstrated a role for ATP-gated K+ channels as well as a role for cyclooxygenase products in mediating propofol-induced depressor responses [43,44]. The extent to which these and other signaling pathways and mediators are involved in mediating the effects of propofol on vascular regulation *in vivo* and *in vitro* will be the focus of future studies. Although CGRP release following A1 and V1 activation is well established, we believe the contribution of CGRP would be minimal. Hexamethonium administration would likely minimise the role for CGRP-induced vasodilation as part of the pre-synaptic transmission would be blocked, however the possibility of post-synaptic TRPA1 activation resulting in CGRP release cannot be discounted. These and is currently the focus of another project in the lab—that of neuronal TRPA1 and CGRP on propofol-mediated vascular relaxation. Regardless, the current study demonstrates for the first time a role for TRPA1 ion channels in mediating propofol-induced vasodepressor responses *in vivo*. The extent to which other anesthetic agents also interact with TRP ion channels to alter vascular function are yet to be determined.
